# 

**Published:** 1890-11-08

**Authors:** 


### NOTICE TO CORRESPONDENTS.

All MS., Letters, Books for Review, and other matters intended for the Editor should be addressed The Editor, The Lodge, Porchester Square, London, W.

The Editor cannot undertake to return rejected M.S., even when accompanied by stamped directed envelope.

**Notice to Advertisers and Subscribers.**

All Advertisements, Orders for Copies of The Hospital, and Business Communications should be addressed to The Publisher, *not to the Editor*, The Hospital, 140, Strand, W.C.

**Bound Volumes of "The Hospital."**

Vol. VI., containing full page portraits of T.R.H. the Prince and Princess of Wales, and Miss Florence Nightingale, is still obtainable. Vol. VII. and all the earlier volumes are also for sale.

Orders will be received for Vol. VIII., 4s. post free. Cases for binding may be had of the Publisher, at 1s. 6d. each.

**To Residents, Secretaries, and Matrons.**

The Editor will be glad to have the Regulations and each Report as issued by Asylums, Hospitals, Convalescent and Nursing Institutions, as well as those of other Charities, and an early copy in duplicate of all Appeals and Festival Notices, etc., addressed to *The Editor, The Lodge, Porchester Square, London, W.* Any superintendent, secretary, matron, or other chief official who regularly sends such information may be registered, on application to the Publisher, to receive free of cost a cover for binding The Hospital in half-yearly volumes.

**BURDETT'S HOSPITAL ANNUAL FOR 1890**

May be obtained of the Publisher, The Hospital Offices, 140, Strand, W.C., price 2s. 6d. limp boards, or 3s. 6d. cloth. All orders must be accompanied by a remittance.

### The Student of Medicine.

[All communications for this Supplement should be addressed to The Editor of The Hospital, at 140, Strand, with the words, "Students' Column," written in the left hand top corner of the envelope.]

### STUDENTS PAST AND PRESENT.

The new session is now in full swing, and all the provincial as well as the London schools have had their introductory addresses and their annual distributions of prizes. Words of welcome and good advice have been showered from many lips upon those two thousand men who have just entered the ranks of the profession. Still more advice and many hints of great practical and theoretical importance have been generously distributed to those beginning their second, third, fourth, and, may be, fifth year; and one and all have been urged on to the goal of medical teachers, the final examination, and with it that token of many books and late hours, namely, the Diploma. The jolly, easy days of the medical student as depicted by many novelists of a generation ago are of the past. A complete change has come about, and the student of medicine of to-day, as compared with one of but a few years back, is well illustrated in the two cartoons published by *Punch* a short time ago. In the one we see Bob Sawyer with his friend Bill Sykes sitting in a room decorated with sporting pictures, chiefly of women, horses, and dogs, weapons of warfare, beer jugs, pipes, knockers, and police apparatus, with a few stray bones thrown in for ornament; they have long pipes in their mouths, feet on the chimney-piece, and a glass of grog by their sides. The other cartoon shows the student of to-day burning the midnight oil, with a damp towel round his head, elbows on the table, face buried in his hands, and head bent forward deeply intent on the pages of a large medical work. Even the fox or bull terrier has disappeared from the diggings, and the yellow-back of uncertain authorship has been replaced by Spencer's Sociology. The love of the bones and tusks of beasts, especially when moulded into round forms by man's device, has been in many quarters superseded by the bones of man and other animals in their natural and unpolished condition.

### GOLIATH.

The giant who is being exhibited in London under this name was shown at a recent meeting of the Pathological Society. He is 39 years of age, a native of Westphalia, and only speaks German. His height is 73½ inches (6ft. 1½ in.), circumference of the head, 26½ in., length, 9 1-5 in., breadth, 6 7-10 in. The hands are enormous, thumb circumference, 4 in., middle finger, length, first phalanx, 4 7-10 in., second phalynx, 3¼ in. He had been of enormous proportions since childhood, and there was no period of his life at which he noticed any unusual rapidity of growth. His muscular development is very great, and he can lift half-a-ton with tolerable ease. The thyroid body is small, and there is no evidence of a persistent thymus. The urine contains sugar. The man is a typical example of that curious disease known as Acromégalie, characterised by an enlargement of the bones, and of the soft parts, of all the extremities. This enlargement is symmetrical, and affects not only the hands and feet, but also the head, especially the bones of the face, thus giving the individual a very extraordinary appearance.

### MEN OF THE FUTURE.

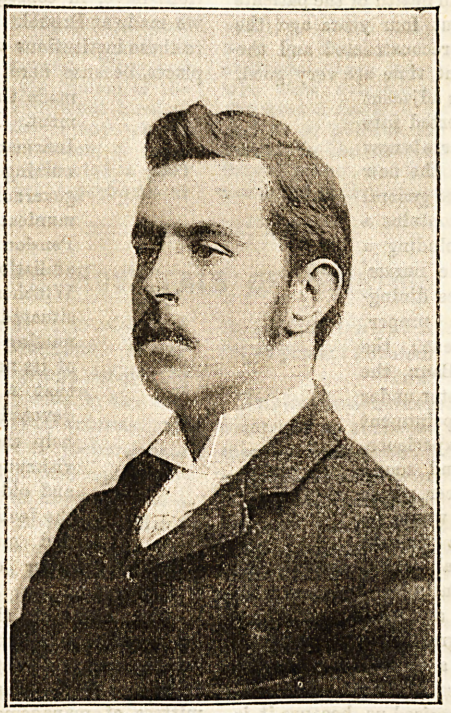

MR. JOHN C. BAKER, B.A. (Barts.)
First Class Honours in Materia Medica in Inter. M.B. London University.

Mr. John Charles Baker, whose portrait we publish with this week's number, took his B.A. degree (1st. div.) in November, 1884, and for a time was a schoolmaster. After attending some classes at St. Bartholomew's Hospital, together with private study in connection with the University Correspondence Classes, he passed the preliminary scientific examination of the London University in the first division in July, 1887. In October of the same year he obtained the Preliminary Scientific Exhibition, and entered St. Bartholomew's Hospital as a medical student. In the following March he passed the first Conjoint College Examination, and the Materia Medica in the July of the same year. The second college was passed in March, 1889, and after this Mr. Baker obtained the Wix prize at the hospital for an essay on the life and works of Dr. Francis Glisson. Mr. Baker has now passed the Intermediate M.B. with first-class honours in Materia Medica and Pharmaceutical Chemistry. We wish him every success in the final examinations with a place well up in the honour list.

### NOTES FROM THE SCHOOLS.

### CHARING CROSS HOSPITAL.

The annual dinner of past and present students of the above hospital recently took place at the Holborn Restaurant, under the presidency of Mr. J. W. Taylor, F.R.C.S., of Birmingham, a former house-surgeon. Between 140 and 150 sat down to dinner, among whom were the following members of the staff and former resident medical and surgical officers: Dr. H. Green, Dr. J. Watt Black, Dr. J. Mitchell Bruce, Dr. A. W. Orwin, Dr. Mott, Messrs. E. Bellamy, J. Astley Bloxam, J. Stanley Boyd, Morgan Woollett, J. B. Cook, P. Delamotte, R. Athill, G. Brown, and G. Davies. In proposing success to the Charing Cross Hospital Medical School, Mr. Taylor referred to the enormous strides which the school had made during recent years, and the great success which had attended the efforts of the staff in raising its reputation to a high position among the medical schools of the metropolis. The dinner was a decided success, a result which was due in a great measure to the efforts of Messrs. Collum and Williams, who acted as honorary secretaries, and to Dr. Mott, who superintended the musical arrangements.

### BRISTOL.

At the Bristol Medical School dinner on Saturday, Mr. J. Greig Smith, in the course of an excellent speech, gave the following interesting account of the life at Bristol in its earliest days, as illustrated by the career of Mr. Thornhill, its first surgeon. He said that in 1735, when the Bristol Royal Infirmary was first founded, Mr. Thornhill was appointed surgeon. In some respects he was the greatest surgeon they had had. He was a man of some repute, was well related, and it was he who first found out the operation as now performed of suprapubic lithotomy. For some reason or other the committee of the Bristol Infirmary thought fit to expel him from the staff, but he refused to leave, and long after he had been expelled he was attending patients there, patients coming from all parts of the district to be treated by him. There was one story of how he fell out with a colleague at the bedside of a patient and gave him a thorough good thrashing. Mr. Greig Smith threw out the excellent suggestion that, as they had three generations of Prichards, and he believed there would be a fourth, and as there were at present two Swaynes, and no name was better known in the medical world of England than that of Joseph Griffith Swayne, they ought to take steps to form a Prichard Museum and a Swayne Library. Other schools had started memorial buildings of this description, and he failed to see why Bristol should not follow so excellent an example. They badly wanted a new medical school building, and he hoped that his suggestion might result in securing ample funds to enable them to complete this necessary work without further delay.

### IRELAND (ST. VINCENT'S HOSPITAL).

The opening address delivered by Surgeon McArdle at St. Vincent's Hospital was an able and spirited one, and the closing sentences dealt most forcibly with the degrading position occupied by army surgeons in the British forces. He described

**Notes from the Schools.—(*Continued.*)**

them justly as being "nonentities having no recognised position and no rank—mere appendages." This must, by one and all, be admitted to be a correct and vivid painting of our army surgeons. Dr. McArdle looks for a remedy when the time arrives in which our examinations shall be made harder to pass, and when the number admitted to our ranks being necessarily less, we shall be in a position to close up our ranks and boldly demand a recognition of our just rights. This is striking the proper keynote, and presents the only and real solution of this problem in particular, and many others in the medical world. So long as the ranks are so abnormally full, so long as candidates can be had for a mere song, so long as the love of greed, position, and power take the place of higher motives in our profession, so long must its members be treated as so many deadheads and semi-pantomimic mummies. Let our licensing bodies have less bartering of degrees, more reality and probity in their examinations, and medicine will once again take its place—its correct position—as the noblest of all professions.

### STUDENTS' MEDICAL SOCIETIES.

### Edinburgh Royal Medical Society.

A very large and overcrowded meeting assembled in the Royal Medical Society's Hall to hear Mr. Jonathan Hutchinson give the inaugural address. Dr. Sanders, President of the Society, was in the chair, and among those present were Professors Grainger Stewart, Chiene, Rutherford, Gairdner, of Glasgow, Drs. Argyll, Robertson, and Littlejohn. Mr. Hutchinson, in commencing, said the difficulty of studying medicine was its uncertainty. Facts in medicine could not be proved by logic or mathematical reasoning. There were many cases in which, although we could give good explanations, it was not in our power to prove them. As an illustration he showed a skull in which there were very large and extensive exostoses on the left side and on that side only. He had no doubt that the trigeminal nerve had by its influence on the nutrition of that part caused this overgrowth of bone, but he could not prove it. There was only one other skull in existence which showed this overgrowth and in an exactly similar manner, and that was in the possession of Dr. Thomson, of Edinburgh. After making a few remarks on tubercle, Mr. Hutchinson went on to discuss leprosy. Leprosy was a disease widely distributed over the world, and it was to be noted that it occurred always on the seashore or on the banks of lakes and rivers. The reason, he believed, was that a considerable amount of fish was eaten in those situations. This was confirmed by the fact that since the Reformation leprosy has decreased, because fasting on salt fish is not enforced. Leprosy is more prevalent where fasting is enforced than where it is not. He believed that leprosy was not contagious, but was due to errors in diet. Again referring to medical research, Mr. Hutchinson said that medical men, as scientific observers, must attend to the laity. The laity were often right. In America the farmers would not allow cranberries to grow in the hedges of their cornfields, as they believed that blight was derived from a fungus of the cranberry. The botanical microscopist declared the fungus of the cranberry was Accidium, and blight was Puccinia graminis. A law was passed prohibiting the destruction of cranberry trees, but it was afterwards found that Accidium and Puccinia were different forms of the same fungus. In concluding, Mr. Hutchinson said that in our studies we needed fixity of attention, and that the study of humanity was essential to success as practitioners. Dr. Sanders moved a vote of thanks to Mr. Hutchinson, which was seconded by Professor Gairdner, of Glasgow. The meeting adjourned.

### SCHOLARSHIPS AND PRIZES.—THIRD LIST.

### St. Bartholomew's Hospital.

Prize Awards.—The following is a list of the prizes, &c., awarded for the sessions, 1889-90. Lawrence scholarship and gold medal, H. A. Powell, Brackenbury medical scholarship, F. Johnson; Brackenbury surgical scholarship, R. C. Bailey; senior scholarship in anatomy, physiology, and chemistry, H. Armstead. Open scholarships in science, chemistry, and physics, W. H. Pollard; biology and physiology, H. J. Paterson; junior, S. S. F. Blackman and F. Fraser, *æq.* Preliminary scientific exhibition: W. E. Lee. Jeaffreson exhibition: R. V. Gilmour. Kirke's scholarship and gold medal: C. E. Williams. Bentley prize (surgical): R. Brown. Hichens prize: B. H. Leumann. Wix prize: J. C. Baker. Harvey prize: J. S. Sloane, D. W. Collings, B. Collyer, S. E. Gill, W. N. Soden. Sir George Burrow's prize: C. R. Stevens. Skynner prize: E. P. Paton. Practical anatomy, junior.—Treasurer's prize: A. C. Gurney, H. B. Meakin, P. E. Adams, S. Cornish, W. Wyllys, R. H. Wilkin, P. C. Barford, G. G. Oakley, F. Fraser, and W. W. W.

### Scholarships and Prizes—(*continued*).

Burges, *æq.*, and W. C. Lee. Practical anatomy, senior.—Foster prize: J. S. Sloane, B. Collyer, W. N. Soden, A. H. Buck, E. S. Humphry, and C. N. Walsh, *æq.*, J. O. March, H. B. Maingay and A. F. Stevens, *æq.*, and J. H. Collyns. Shuter scholarship: J. Attlee. Junior scholarships: A. C. Gurney and F. Fraser. Junior scholarships in chemistry (1889): W. N. Soden, and B. Collyer and H. A. Andrews, *æq.*

### PASS LISTS.

### Conjoint Scheme of the College of Physicians and Royal College of Surgeons in Ireland.

The under-mentioned gentlemen have passed the Second Professional Examination ;—

M. S. Bell, M. Birch, A. Burke, E. Cairns, E. R. Cotter, O. Elsner, G. Fleming, F. Golding, J. V. Griffin, J. Harold, T. C. Harte, R. Hayden, H. Hayes, E. Hunt, H. Hunt, P. Monahan, P. Murray, W. McCann, J. G. McLannahan, F. W. Perry, W. Pigott, C. Richards, R. Smith, J. Woodside.

The following candidates have passed in one or more of the subjects in which they presented themselves :—

H. Beasley, W. Burke, E. Chartres, C. Comber, W. Coneys, T. Cotter, J. Crozier, M. Cuffe, T. C. Cummins, R. Fisher, J. Fox, R. Harding, T. A. Hartigan, H. Jacob, R. Jephson, W. Lappin, H. B. Ludlow, J. F. Maguire, A. J. Moran, C. Murphy, W. O'Donnell, M. J. McMahon, J. F. Sheppard, C. Skelly, E. Smith, E. Stubbs, V. B. Taylor, and A. R. Twigg.

### Royal University in Ireland.

The following gentlemen have passed the Medical Degrees Examination of the University :—

UPPER PASS DIVISION.—John Adams, James Brown, *Martin Dempsey, George Frost, *Andrew Fullarton, *T. W. Fullarton, James Keegan, James Moore. *Allowed to compete for Honours.

PASS.—Joseph Anderson, Edgar Cane, William Cluff, Patrick Crowley, John E. Cruise, Maurice Fitzgerald, T. H. Foley, Robert Fraser, James Harrison, James Henney, O. P. Hill, Thomas Jackson, Henry Jamison, John Jones, Bertrand Jordan, R. J. Keane, Robert Lyons, James Mc Mordie, Michael Mahoney, John Mills, James Morrow, John Nevin, Hugh Stranaghan, Samuel Tate, Edward Tierney, A. J. Tonkin, George Walker, and T. B. Wilkinson.

### Victoria University, Manchester, October 30th.

EXAMINATION LISTS; FACULTIES OF ARTS AND SCIENCE.

INTERMEDIATE B.A., EXAMINATION.—J. E. Bamford, Owen's College; Louisa Gibson, University College; J. A. Jones, Owen's College; Samuel Tindall, Owen's College; Margaret E. Watson, Owen's College; Joseph Wood, Owen's College.

FINAL B.A. EXAMINATION, SECOND DIVISION.—P. E. Ireland, Owen's College.

FINAL B.Sc. EXAMINATION, FIRST DIVISION.—T. B. Cooper, Owen's College; R. M. M'Dougall, Owen's College; E. H. Rigby, University College. Second Division—W. E. Wild, Owen's College.

### Glasgow.

The following gentlemen have passed the First Professional Examination for the degrees of Bachelor of Medicine (M.B.) and Master in Surgery (C.M.) :—

R. Y. Aitken, J. D. Bauchop, D. Beatson, W. Blair, R. Boyd, T. S. Brodie, J. P. Brown, M.A., J. Brown, T. D. Brown, A. J. Brownlee, R. Bruce, A. Cameron, E. A. Campbell, J. J. Carruthers, H. B. Causley, V. E. Chang, J. M'N. Christie, J. Clark, W. S Cook, J. Cumming, A. Dingwall, M.A., D. Dingwoodie, F. Ditmar, M.A., A. Dodds, A. Donald, W. Donaldson, J. E. Downs, F. Elliott, J. Ferguson, J. W. Findley, A. Forrest, R. W. Forrest, G. W. Francis, J. Fullarton, W. Fyfe, J. F. R. Gairdner, J. G. Gitson, T. B. Gilchrist, R. K. Goldie, H. B. Grant, R. Grieve, M.A., D. F. Harris, C. B. Harrison, A. G. Hay, M.A., G. S. Illingworth, G. Innes, M.A., W. Irving, J. R. Jeffrey, W. L. Jones, T. Kay, A. J. Laird, R. Langmuir, M.A., J. F. Lees, R. R. Manners, C. S. Marshall, T. B. Marshall, J. Masterton, D. M'F. Millar, B. B. Morton, T. R. Muir, P. McBride, M'Callum, J. M'Feat, A. McGregor, W. A. Mackay, J. M'Kie, J. I. Macmillan, M.A., T. M'Nay, B. S. Nicholson, G. Nicoll, J. H. Ormond, A. C. Park, J. Patrick, M.A., A. F. S. Pearcey, E. L. Pollonais, W. P. Porter, A. Rachman, J. Rankin, W. Rankine, A. Revie, A. S. Robertson, A. M'L. Robertson, J. Robertson, J. C. Robertson, S. K. Rey, J. Shaw, A. N. Sinclair, E. P. Sinclair, M. A., M'I. Sinclair, W. S. Sinclair, J. M. Smith, J. Sturrock, E. Sugden, J. Thomas, J. R. Turner, J. J. Urwin, F. H. Waddy, A. A. Warden, M.A., J. L. Watson, R. G. Whitelaw, J. Y. Whyte, H. W. Williams, J. D. O. Wilson, H. R. Wolfe, R. T. Wood, S. M'K. Wotherspoon, A. G. Young, A. Young.

The following gentlemen have passed the Second Professional Examination :—

R. H. Allen, C. J. Babes. T. Barrowman, J. E. Bow, J. J. Boyd, W. B. Brodie, F. Brown, W. M. Brown, A. Cameron, D. G. Carmichael, R. J. Carroll, J. Carslaw, M.A., W. Cassels, J. Cochrane, D. A. Dewar, P. F. Dewar, A. S. Dick, T. Divine, W. W. Don, G. A. Eadie, E. A. Eckersley, A. R. Ferguson, A. Findlay, R. Forsyth, J. J. Fraser, W. Fulton, T. A. S. Gibb, D. M'I. Glen, F. Gracie. R. Guy, T. Hamilton, J. B. Hartley, E. D. S. Heyliger, A. Higgie, C. Highet, J. L. Howie, G. L. Kerr, H. Kerr, M.A., T. Kirkwood, M.A., T. D. Laird, J. Lamont, J. Lindsay, J. B. Littlejohn, W. Lowe, H. C. Marr, J. R. M'C. Millar, M.A., J. Moffatt, J. D. R. Monro, M.A.; J. Morton, J. H. MacArthur, W. M'Call, J. M'Donald, M.A., G. M'Feat, J. M'Kay (Dumbarton), M. Macnicol, M.A., A. S. M'Pherson, P. C. MacRobert, W. M'Walter, R. E. Newton, J. Paterson, M.A., J. Pearson, W. J. Richard, M.A., J. J. Robb, H. Robertson, J. A. Robertson, L. A. Rowden, T. L. Shields, J. Smyth, W. Steel, J. B. Stevens, C. Stewart, R. Taylor, J. H. Teacher, M.A.,

**Pass Lists**—(*continued*).

J. P. Thomson, D. Wallace, A. Wauchope, A. White, C. Wilson, T. Wright, A. A. Young, M.A., J. M. Young, and W. S. Young.

The following gentlemen have passed the Third Professional Examination, including, in the case of those marked with an asterisk, the subject of Pathology:—

### ATHLETICS.

### Football.

The following matches were played on November 1st:—

*St. Bartholomew's Hospital* v. *Rangers*.—Raynes Park was the venue of the Cup tie in the second round of the London qualifying competition between the above-mentioned clubs, when the Hospitallers simply made rings round their opponents, and ultimately left the field victorious by eight goals to nil.

*Old Leysians* beat *Cambridge University*, at Cambridge, in beautiful weather. Both clubs were well represented, and an exceedingly fast game ensued. The University pressed their opponents at the start, and Forde scored between the posts. After fifteen minutes' play Wotherspoon placed a goal. The Old Boys then retaliated, and before half-time Brooke and J. W. M'Arthur scored tries, neither of which were improved upon. In the second half the University hardly had a look in, the visitors being within an ace of scoring a dozen times; however, they only obtained two tries, through the agency of Gordon Beves and Montagu Beves. The place-kick was not successfully undertaken in either instance. The Old Leysians finally won by four tries to one goal. Referee, W. H. Denman.

*Oxford University* beat *R.I.E.C., Coopers' Hill*, at Oxford, in lovely weather, before about 2,000 spectators. Fleming kicked off at five minutes past three, and a stubborn fight ensued for the first half of the game, the interval arriving with the score—Oxford, one try; Coopers' Hill, nil. With change of ends, however, the home team were seen to great advantage, the forwards playing with plenty of dash, whilst the passing between the halves and three-quarters carried all before it; and when the whistle finally sounded, the Dark Blues had gained a decisive victory by two goals and three tries to nil. Fleming was responsible for three tries, and Bonham Carter and Clauss were accredited with one each; whilst Cochran placed the goals. Sutherland played a sterling game at half-back, and Todd Morris, P. M. Latham, Humphries, and Willmott all worked hard for the losers.

*King's College (Second)* beat *University (Second)* at Wormwood Scrubs, by ten goals to one. *Kingston* beat *Charing Cross Hospital*, at Kingston, by two goals and one try to nil. *Mitcham* beat *St. Bartholomew's Hospital (Second)* by a goal and a try to a goal. *Lewisham Park* beat *Middlesex Hospital (A)* by one goal and two tries to nil. *Rosslyn Park* beat *Guy's (Second)* by one goal and four tries to one try. Tries by H. Welby (two), E. Welby, Sharman, and Hayle (one each). *Crusaders* beat *Cambridge University*.—The 'Varsity played their third match at Cambridge before a large crowd, and although they counted several old Blues in their team, they were unable to cope with the visitors, who gained an easy victory by four goals to one. *St. Mary's Hospital* beat *Thornton Heath* by three goals to nil. *St. George's Hospital* beat *Windsor* by two goals to nil. *Clapham Park* beat *Westminster Hospital*, at Raynes Park, by two goals to nil. (R.) *Old Dovorians* beat *Middlesex Hospital*, at Balham, by one goal and one try to one goal. (R.) *St. Thomas's Hospital* beat *Richmond*, at Richmond, by two goals and three tries to nil. (R.) *Swansea* beat *Guy's Hospital*, at Swansea, by two tries to nil. (R.) *Sarcens* and *London Hospital* at Walthamstow, drawn, nil to nil. (R.) *Crusaders* beat *Cambridge University*, at Cambridge, by four goals to one. (A.) *St. Thomas's Hospital* beat *Chiswick Park*, at Upper Tooting, by three goals to one. (A.)

*Owen's College* v. *Rochdale Hornets*. — This match was played on Wednesday, October 29th. The college team was rather weak in the back division, owing to the absence of Hall at three-quarters. The game was very evenly contested during the first half of the time, but in the last quarter the Hornets crossed the college lines three times. Score, Rochdale Hornets, 1 goal, 2 tries, 3 minors to nil.

### EVENTS FOR THE MONTH OF NOVEMBER.

### November Meetings and Athletics.

For list of Students' Medical Societies' Meetings and Football Matches during November, see THE HOSPITAL for Nov. 1st, p. 84.

**The Hospital Advertiser.**

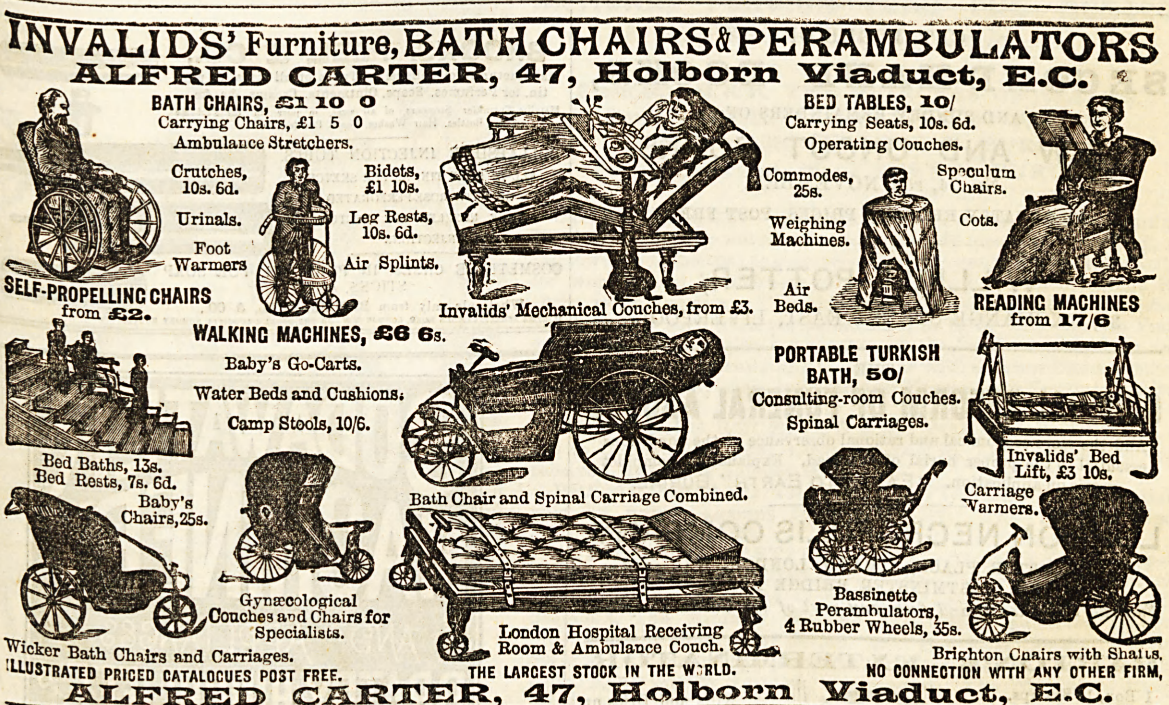

Keen's Mustard
THE OLDEST MANUFACTORY IN THE CITY OF LONDON

Silks, Dresses, Mantles, Costumes, &c.
Peter Robinson.
Oxford St. Regent St.

### General Charities.

[This Column is devoted to an account of work of charitable institutions other than Medical Charities. The Editor will be glad to receive reports or news of work at 140, Strand. *Philanthropy* should be plainly written on the envelope.]

A dramatic recital of "Macbeth" will be given by Mr. E. Glossop Such, assisted by members of the Shakespeare Reading Society, on Tuesday, the 11th inst., at the Portman Rooms, Baker Street, at eight p.m., in aid of the **HOME OF REST FOR HORSES**. Tickets can be obtained at the office of the Home, 185, Victoria Street, S.W.; at the Portman Rooms; or through Mr. E. Glossop Such, 19, Loudoun Road, N.W.

**THE GOVERNESSES' BENEVOLENT INSTITUTION** which has for its objects the relief of governesses in temporary difficulty, the granting of annuities to aged governesses, the securing of deferred annuities to governesses upon their own payments, a home for those who are disengaged, free registration, a college, with classes and certificates of qualification which was separately incorporated nearly forty years ago, and an asylum for aged governesses is to be congratulated on its flourishing condition. The Board of Management announces that the past year has been productive of more annual subscriptions than for many years previously, notwithstanding the loss by death of numerous staunch supporters. The system of subdividing the operations of the institution into various branches permits of subscribers devoting their donations to any particular fund which they may prefer. Those who consider mere monetary assistance degrading to the class can support the provident fund, or the annuity fund, or the asylum at Chislehurst for aged governesses. The provident fund has been a very successful branch of the institution, the amount invested by governesses in it being over £552,000. It includes a savings bank, in which each lady's money stands in her own name and from which the money can only be drawn by her order. That the fund should have attained such dimensions speaks well for the spirit of self-help which leads so many of a poor class of society to put by for the future, and the public must feel that every penny contributed to the furtherance of the object of this branch is money well bestowed. The premiums received for these annuities are paid to the National Debt Office in the names of the annuitants, and therefore the society gains no benefit from them; any money given by subscribers helps both to enlarge the fund and to pay the expenses attendant on the management of the fund. The board of management have, by means of the generous support given them on previous occasions, been able to reduce by one-fourth the payments which were made by ladies for deferred annuities in all cases in which the help was desired, and they now appeal for further contributions in the hope that they may be enabled to continue to reduce the first premiums of those who shall commence paying for annuities. Under the tables which have been drawn up, a lady of twenty-five years of age contracting for an annuity of £30, to commence at the expiration of twenty-five years, would pay an annual premium of £13 17s. 6d., but it has become possible to reduce the first payment to £10 8s. 2d. It sometimes happens that a lady is unable to continue the yearly payments, in such a case all previous payments are returned to her, on three months' notice, without any abatement, but without interest. Thus, if the worst happens no intending annuitant need fear that she is sinking her money in vain. Should death occur before the annuity is payable, the whole of the payments will be returned to the heirs. Offices, 32, Sackville Street, W.

It appears from the latest report of the **PORTSMOUTH SEAMEN'S AND MARINES' ORPHANAGE**, that the financial position of the Schools and Home is not quite so satisfactory as the friends of the Institution would wish it to be. The receipts during the past year have been so far short of the expenditure as to necessitate an appeal to the public, on whom the Institution, which does really practical work has an undoubtful claim, to come forward and help the managers to keep out of debt. The chief supporters of such a charity should, however, be the officers, seamen, and marines of the fleet; but the nature of the Society's work justifies an appeal for help from a wider section of the public. At the annual meeting held last month, it was pointed out by one speaker that as nine per cent. of the girls had gone out from the Institution as thoroughly trained and efficient domestic servants, its claims on the public are undeniable. These claims might have been enforced with greater effect had the speaker stated what was the result of the training of the society in the remaining 91 per cent. of the girls. Up to the present time lessons in cooking seem to have been omitted from the instruction imparted to the girls. Their addition should enable the institution to find occupation for a still larger number of its pupils. Of the 162 girls in the home during the past twelve months, 102 were on the funds of the institution at Portsmouth, and sixty on those of Greenwich Hospital. Fifteen had been placed in service, and four were withdrawn on their friends representing that they were able to support their children. The operations of the society are not confined to girls; there is also a school for boys, who during the same period numbered 67. It is hoped that a home may be found for the boys in the country away from their present bad surroundings. The moral benefit which will be derived from such a change will well repay the most generous support of the public, without whose help the society will not find it easy to accomplish this desirable end.

